# Preoperative systemic immune-inflammation index predicts prognosis of patients with non-metastatic renal cell carcinoma: a propensity score-matched analysis

**DOI:** 10.1186/s12935-020-01320-w

**Published:** 2020-06-08

**Authors:** Xu Hu, Yan-Xiang Shao, Zhi-Qiang Yang, Wei-Chao Dou, San-Chao Xiong, Xiang Li

**Affiliations:** 1 West China School of Medicine/West China Hospital, Sichuan University, Chengdu, 610041 People’s Republic of China; 2 Department of Urology, West China Hospital, West China Medical School, Sichuan University, 37 Guoxue Street, Chengdu, 610041 People’s Republic of China

**Keywords:** Systemic immune-inflammation index, Renal cell carcinoma, Non-metastatic, Prognosis

## Abstract

**Background:**

A novel systemic immune-inflammation index (SII), based on the neutrophils, lymphocytes and platelet counts, is associated with the prognosis of several cancers. The present study evaluates the prognostic significance of SII in non-metastatic renal cell carcinoma (RCC).

**Method:**

The present study retrospectively reviewed the medical record of patients with non-metastatic RCC who underwent nephrectomy between 2010 and 2013. Receiver operating characteristic (ROC) curve analysis was performed to identify the optimal cut-off value. In addition, the propensity score matching (PSM) was performed with a matching ratio of 1:1. Univariate and multivariate Cox proportional hazards models were used to identify the prognostic factors. The results were reported by hazard ratio (HR) with 95% confidence interval (95% CI).

**Results:**

A total of 646 patients were included in the final analysis. High SII group (> 529) was significantly associated with older age (P = 0.014), larger tumor (P < 0.001), higher pathological T stage (P < 0.001), higher tumor grade (P < 0.001) and more tumor necrosis (P < 0.001). Multivariate Cox regression analysis demonstrated that the higher preoperative SII was significantly associated with worse overall survival (OS) (HR = 2.26; 95% CI 1.44–3.54; P < 0.001) and cancer-specific survival (CSS) (HR = 2.17; 95% CI 1.33–3.55; P = 0.002). After PSM, elevated preoperative SII was an independent predictor of poor OS (HR = 1.78; 95% CI 1.1–2.87; P = 0.018) and CSS (HR = 1.8; 95% CI 1.07–3.03; P = 0.027).

**Conclusion:**

In conclusion, preoperative SII is associated with adverse factors for RCC. Furthermore, higher preoperative SII is an independent predictor of poor OS and CSS in surgically treated patients with non-metastatic RCC. More prospective and large scale studies are warranted to validate our findings.

## Background

Renal cell carcinoma (RCC) is one of the most common urological cancers and represents an increased global burden on human healthcare [1]. RCC accounts for approximately 2–3% of all malignancies, with an estimated 403,262 new cases and [1] 75,098 new deaths worldwide in 2018 [2]. Surgical resection is the curative treatment for localized RCC [3]. However, reportedly, about 20–30% of patients will develop recurrence [4, 5]. Nowadays, several treatment strategies have developed well, such as immunotherapy, radiotherapy and molecular target drugs, and the clinical outcomes of the advanced disease have been improved [3, 6, 7]. However, the clinical outcomes remain not encouraging due to the low objective response rate, local recurrence or distant metastases. Therefore, identifying the prognostic factors of patients would be of great value to patients’ risk stratification, treatment selection, and long-term outcomes.

TNM stage, tumor grade, and histology are commonly used prognostic factors, however, these factors would lack information about patient-related factors. Increasing evidence suggested that host inflammation response plays an important role in cancer development and progression [8, 9]. The complement system also plays an important role in the innate immune system. The complement system could recognize invading pathogens and eliminates them by several effector mechanisms that may also cause systemic inflammatory response, as well as using direct lytic destruction [10]. Besides, complement could be leveraged by neoplasms to promote tumorigenic microenvironments and antibody-dependent complement activity is associated with antitumor efficacy [11]. Previous reports have shown that circulating immune-inflammatory cells, such as lymphocytes, neutrophils, and platelet, could contribute to promoting cancer cell proliferation and invasion [8, 9, 12, 13]. Several studies have evaluated these circulating cells and found that they are associated with the prognosis of malignancies [13, 14].

A novel systemic immune-inflammation index (SII), based on the neutrophils, lymphocytes and platelet counts, has been found to be associated with the prognosis of several cancers, such as gastric cancer, hepatocellular carcinoma, bladder cancer as well as RCC [15–18]. However, the prognostic value of SII in non-metastatic RCC is not well explored. Hence, the present study was performed to evaluate the prognostic significance of preoperative SII in non-metastatic RCC patients who underwent nephrectomy.

## Materials and methods

### Patients

After approval by the institutional review board of Sichuan University West China Hospital, the present study retrospectively reviewed the medical record of patients with non-metastatic RCC who underwent nephrectomy between 2010 and 2013. Patients with multiple tumors, presence of inflammation, insufficient information, positive lymph node, and distant metastasis were excluded. At last, a total of 646 patients were included in the final analysis.

The following clinicopathological characteristics were retracted from medical records: age, gender, hypertension, diabetes mellitus, tumor laterality, surgical approach and type, tumor stage, grade, neutrophils, lymphocytes, and platelet counts. All laboratory tests were performed within 1 week before surgery. SII was calculated based on the following formula: platelet × neutrophil/lymphocyte. In addition, the X-ray or computed tomography (CT) scan of the chest and CT or magnetic resonance imaging (MRI) scan of the abdomen should have been performed for patients to identify the preoperative stage. Lymph node dissection was conducted in patients with enlarged nodes on preoperative imaging or palpable nodes during surgery. Tumor size was recorded as the longest tumor diameter based on the pathological report. The pathological stage was classified based on the 8th edition of the tumor-node-metastasis (TNM) classification [19]. The pathological evaluation was made according to the 2016 WHO classification [20].

### Follow-up data

All patients were followed up regularly, every 3 months within 2 years after surgery, every 6 months for the next 3 years, and then once a year. Patients should undergo a medical history, physical examination, laboratory test, X-ray or CT of the chest, ultrasound or CT or MRI of the abdomen. The clinical outcomes include overall survival (OS) and cancer-specific survival (CSS). OS is defined as the interval time between the day of surgery and the last follow-up or all-cause death. CSS is measured from the day of surgery and last follow-up or cancer-related death.

### Statistical analysis

Continuous variables are presented as mean ± standard deviation (SD) or median (interquartile range, IQR). Categorized variables are described as frequency (percentage). Chi square test and Student’ t test were used to compare the categorized and continuous variables, respectively. Receiver operating characteristic (ROC) curve analysis was performed to identify the sensitivity and specificity of the prognostic factor. The optimal cut-off value of the prognostic factor was chosen based on the Youden Index. Kaplan–Meier survival curve and the log-rank test were carried out to compare the survival outcomes between groups. Univariate and multivariate Cox proportional hazards models were used to identify the prognostic factors of OS and CSS. The results were reported by hazard ratio (HR) with 95% confidence interval (95% CI).

In addition, the propensity score matching (PSM) was performed using the nearest neighbor method with 0.02 calibration adjusting for preoperative characteristics. The matching ratio is 1:1, and SII (low vs high) is the dependent variable. The standardized difference was calculated to evaluate the covariate balance, with a value < 0.10 regarded as balanced [21]. A two-tailed *P* value < 0.05 was regarded as statistically significant. All statistical analyses were performed by R software version 3.6.2 (http://www.r-project.org/) and IBM SPSS Statistics version 23.0 (IBM Corp, Armonk, NY).

## Results

### Clinical characteristics of patients

The clinical characteristics of the included patients were summarized in Table 1. The mean age of the patients was 54.77 years (SD ± 12.61). The final cohort included 394 men (60.99%) and 252 women (39.01%) with a mean tumor size of 4.97 cm (SD ± 2.53). More than half of the patients received open surgery (69.81%) and radical nephrectomy (67.49%). Most patients (n = 543, 84.06%) had clear cell RCC. Pathological T stage was T1 in 522 cases (80.80%), T2 in 53 (8.2%), T3 in 63 (9.75%), and T4 in 8 (1.24%). The median follow-up was 84 months (IQR, 75–93 months).

**Table 1:** Clinical characteristics of the patients

	Total	Before PSM			After PSM		
		SII			SII		
		< 529	> 529	P-value	< 529	> 529	P-value
No. of patients	646	483	163		163	163	
Age (years)	54.77 ± 12.61	54.03 ± 12.52	56.95 ± 12.68	0.014	54.93 ± 12.84	56.95 ± 12.68	0.214
Gender				0.159			0.816
Male	394 (60.99%)	287 (59.42%)	107 (65.64%)		105 (64.42%)	107 (65.64%)	
Female	252 (39.01%)	196 (40.58%)	56 (34.36%)		58 (35.58%)	56 (34.36%)	
Hypertension	169 (26.16%)	114 (23.60%)	55 (33.74%)	0.011	44 (26.99%)	55 (33.74%)	0.185
Diabetes mellitus	77 (11.92%)	51 (10.56%)	26 (15.95%)	0.066	18 (11.04%)	26 (15.95%)	0.195
Laterality				0.203			0.506
Left	313 (48.45%)	227 (47.00%)	86 (52.76%)		80 (49.08%)	86 (52.76%)	
Right	333 (51.55%)	256 (53.00%)	77 (47.24%)		83 (50.92%)	77 (47.24%)	
Tumor size (cm)	4.97±2.53	4.57±2.13	6.16±3.16	< 0.001	5.85±2.71	6.16±3.16	0.641
Operative approach				0.305			0.705
Open	451 (69.81%)	332 (68.74%)	119 (73.01%)		122 (74.85%)	119 (73.01%)	
Laparoscopic	195 (30.19%)	151 (31.26%)	44 (26.99%)		41 (25.15%)	44 (26.99%)	
Nephrectomy				< 0.001			0.597
Radical	436 (67.49%)	308 (63.77%)	128 (78.53%)		124 (76.07%)	128 (78.53%)	
Partial	210 (32.51%)	175 (36.23%)	35 (21.47%)		39 (23.93%)	35 (21.47%)	
Pathological T stage				< 0.001			0.486
T1	522 (80.80%)	413 (85.51%)	109 (66.87%)		111 (68.10%)	109 (66.87%)	
T2	53 (8.20%)	32 (6.63%)	21 (12.88%)		28 (17.18%)	21 (12.88%)	
T3	63 (9.75%)	34 (7.04%)	29 (17.79%)		21 (12.88%)	29 (17.79%)	
T4	8 (1.24%)	4 (0.83%)	4 (2.45%)		3 (1.84%)	4 (2.45%)	
Histologic subtype				0.807			0.636
Clear cell	543 (84.06%)	405 (83.85%)	138 (84.66%)		141 (86.50%)	138 (84.66%)	
Non-clear cell	103 (15.94%)	78 (16.15%)	25 (15.34%)		22 (13.50%)	25 (15.34%)	
Tumor grade				< 0.001			0.145
G1	24 (3.72%)	20 (4.14%)	4 (2.45%)		3 (1.84%)	4 (2.45%)	
G2	340 (52.63%)	276 (57.14%)	64 (39.26%)		72 (44.17%)	64 (39.26%)	
G3	263 (40.71%)	181 (37.47%)	82 (50.31%)		84 (51.53%)	82 (50.31%)	
G4	19 (2.94%)	6 (1.24%)	13 (7.98%)		4 (2.45%)	13 (7.98%)	
Tumor necrosis	71 (10.99%)	38 (7.87%)	33 (20.25%)	< 0.001	32 (19.63%)	33 (20.25%)	0.890
Sarcomatoid differentiations	7 (1.08%)	3 (0.62%)	4 (2.45%)	0.072	2 (1.23%)	4 (2.45%)	0.685

**Fig. 1:**
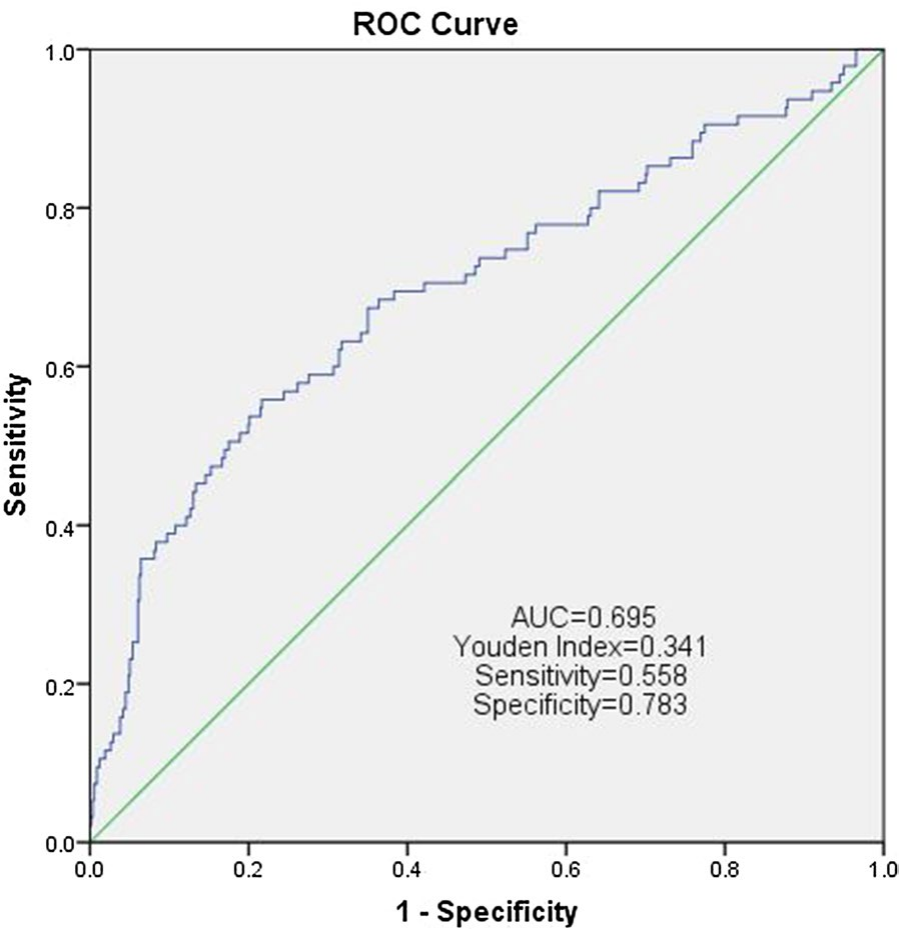
ROC curve analysis of CSS for RCC patients

The optimal cut-off value of SII is 529 based on the maximum Youden index (Fig. 1). Thus, the patients were divided into two groups. The patients in high SII group (> 529) were significantly associated with older age (P = 0.014), larger tumor (P < 0.001), higher pathological T stage (P < 0.001), higher tumor grade (P < 0.001) and more tumor necrosis (P < 0.001), compared with those in low SII group (< 529). After PSM, 326 patients were identified, and there was no significant difference in baseline between low and high SII group.

### Association between preoperative SII and survival outcomes before PSM

**Fig. 2:**
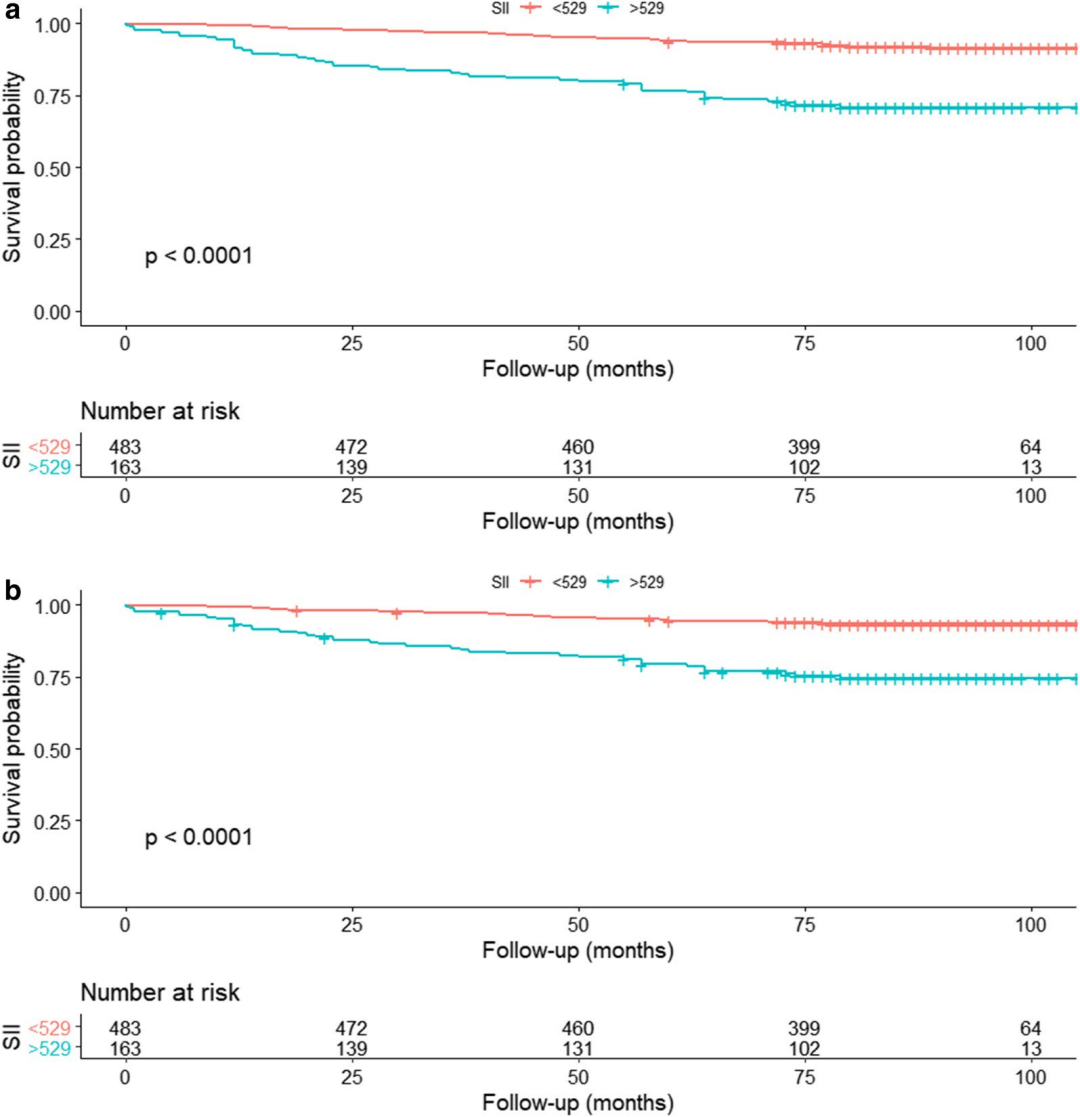
Association between SII and **a**: OS and **b**: CSS in non-metastatic RCC patients after nephrectomy before PSM

After a median follow-up of 84 months, 85 patients (13.16%) had died and 71 deaths (10.99%) were related to RCC. The 5-year OS rates were 93.79% and 76.67% for the patients in low SII and high SII groups, respectively. The 5-year CSS rate was 94.39% for the low SII group, 79.38% for the high SII group. Kaplan–Meier survival curve showed that the low SII had a better OS (P < 0.001; Fig. 2a) and CSS (P < 0.001; Fig. 2b) than the high SII group. Besides, the multivariate Cox regression analysis demonstrated that the higher preoperative SII was significantly associated with worse survival outcomes in terms of OS (HR = 2.26; 95% CI 1.44–3.54; P < 0.001) and CSS (HR = 2.17; 95% CI 1.33–3.55; P = 0.002). Furthermore, age, surgical approach and type, T stage, tumor grade, tumor necrosis, and sarcomatoid feature were also the independent predictors of prognosis (Table 2).

**Table 2:** Univariate and Multivariate Analyses of Prognostic Factors for OS and CSS in patients with non-metastatic RCC (n = 646)

	OS		Multivariate		CSS		Multivariate	
	Univariate				Univariate			
	HR (95% CI)	P-value	HR (95% CI)	P-value	HR (95 CI)	P-value	HR (95% CI)	P-value
Age (≥ 55 vs < 55)	2.9 (1.79–4.72)	< 0.001	1.77 (1.07–2.92)	0.025	2.39 (1.44–3.98)	0.001		
Gender (male vs female)	1.19 (0.76–1.86)	0.437			1.28 (0.78–2.09)	0.332		
Surgical approach (laparoscopic vs open)	0.29 (0.15–0.56)	< 0.001	0.41 (0.21–0.81)	0.009	0.24 (0.11–0.52)	< 0.001	0.36 (0.16–0.79)	0.011
Type of surgery (partial vs radical)	0.17 (0.08–0.37)	< 0.001	0.37 (0.17–0.83)	0.016	0.11 (0.04–0.31)	< 0.001	0.29 (0.1–0.82)	0.019
Histology (non-clear cell vs clear cell)	0.96 (0.53–1.74)	0.896			1.08 (0.58–2.01)	0.807		
T stage (3–4 vs 1–2)	8.9 (5.78–13.7)	< 0.001	3.99 (2.5–6.37)	< 0.001	12.4 (7.77–19.8)	< 0.001	5.25 (3.16–8.7)	< 0.001
Tumor grade (G3–4 vs G1–2)	4.4 (2.69–7.21)	< 0.001	1.83 (1.06–3.14)	0.03	5.83 (3.25–10.5)	< 0.001	2.15 (1.14–4.08)	0.019
Tumor necrosis (yes vs no)	5.29 (3.39–8.26)	< 0.001	2.38 (1.49–3.79)	< 0.001	6.2 (3.85–9.99)	< 0.001	2.65 (1.62–4.36)	< 0.001
Sarcomatoid feature (yes vs no)	10.66 (4.3–26.4)	< 0.001	2.92 (1.14–7.45)	0.025	12.6 (5.06–31.5)	< 0.001	3.72 (1.44–9.6)	0.007
SII (> 529 vs < 529)	4.23 (2.76–6.49)	< 0.001	2.26 (1.44–3.54)	< 0.001	4.38 (2.74–7.01)	< 0.001	2.17 (1.33–3.55)	0.002

### Association between preoperative SII and survival outcomes after PSM

**Fig. 3:**
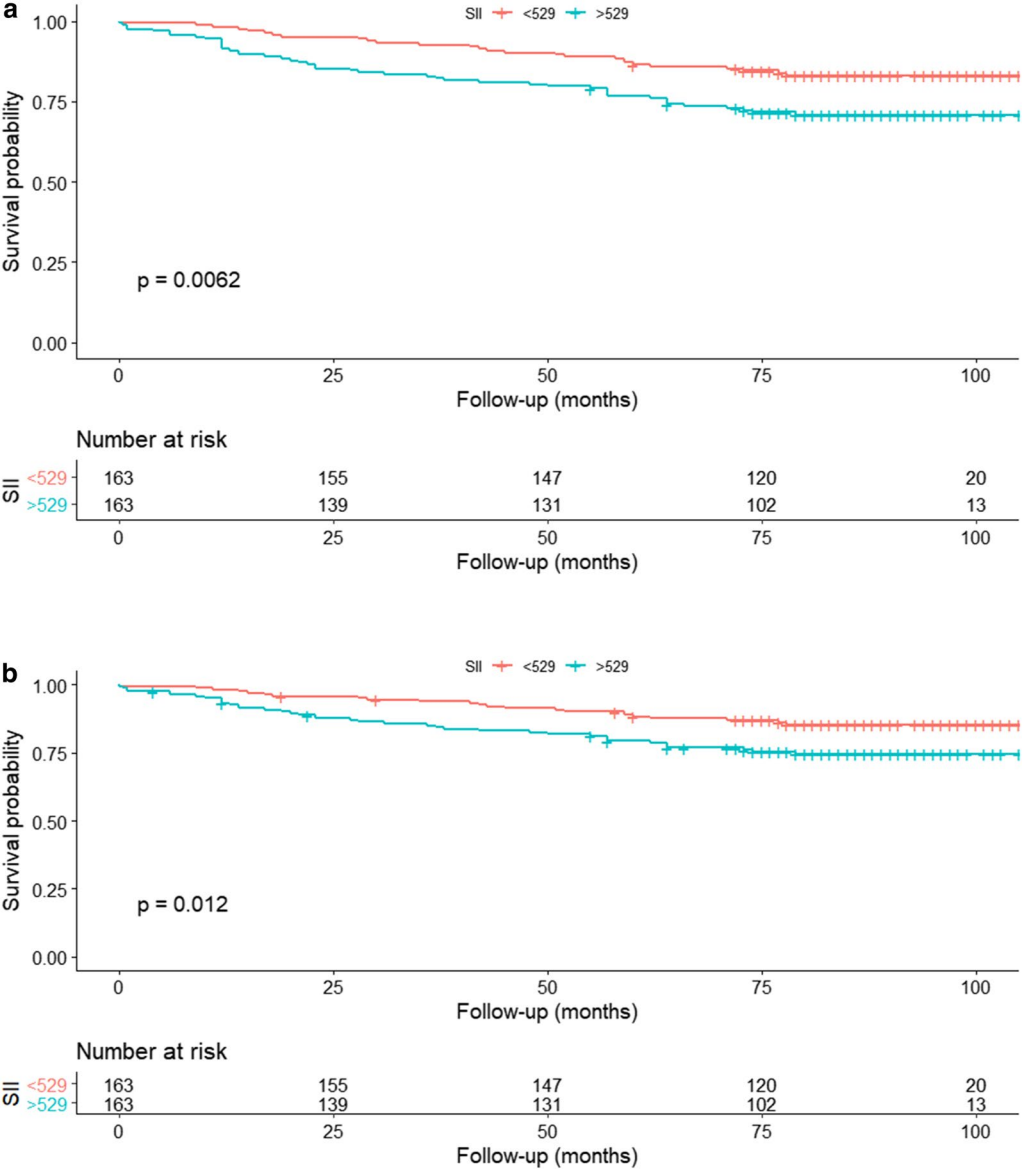
Association between SII and **a**: OS and **b**: CSS in non-metastatic RCC patients after nephrectomy after PSM

After a median follow-up of 81 months, 74 patients (22.70%) had died and 63 deaths (19.33%) were related to RCC. The 5-year OS rates were 86.50% and 76.67% for the patients in low SII and high SII groups, respectively. The 5-year CSS rate was 88.24% for the low SII group, 79.38% for the high SII group. Kaplan–Meier survival curve indicated that higher preoperative SII was associated with lower OS (P = 0.006; Fig. 3a) and CSS (P = 0.012; Fig. 3b). Based on the multivariate analysis, the elevated preoperative SII was an independent predictor of poor prognosis regarding OS (HR = 1.78; 95% CI 1.1–2.87; P = 0.018) and CSS (HR = 1.8; 95% CI 1.07–3.03; P = 0.027; Table 3).

**Table 3:** Univariate and Multivariate Analyses of Prognostic Factors for OS and CSS in patients with non-metastatic RCC after PSM (n = 326)

	OS		Multivariate		CSS		Multivariate	
	Univariate				Univariate			
	HR (95% CI)	P-value	HR (95% CI)	P-value	HR (95% CI)	P-value	HR (95% CI)	P-value
Age (≥ 55 vs < 55)	2.06 (1.25–3.4)	0.004			1.74 (1.03–2.94)	0.038		
Gender (male vs female)	1.16 (0.71–1.89)	0.550			1.39 (0.81–2.41)	0.234		
Surgical approach (laparoscopic vs open)	0.31 (0.15–0.65)	0.002	0.42 (0.2–0.87)	0.021	0.22 (0.09–0.55)	0.001	0.32 (0.13–0.8)	0.015
Type of surgery (partial vs radical)	0.17 (0.06–0.46)	0.001	0.31 (0.11–0.88)	0.028	0.15 (0.05–0.47)	0.001		
Histology (non–clear cell s clear cell)	0.71 (0.34–1.48)	0.365			0.86 (0.41–1.8)	0.682		
T stage (3–4 vs 1–2)	6.4 (4.04–10.14)	< 0.001	4.09 (2.49–6.71)	< 0.001	8.91 (5.4–14.7)	< 0.001	5.39 (3.14–9.25)	< 0.001
Tumor grade (G3–4 vs G1–2)	2.79 (1.64–4.75)	< 0.001			3.81 (2.03–7.14)	< 0.001		
Tumor necrosis (yes vs no)	2.91 (1.83–4.65)	< 0.001	2.15 (1.33–3.48)	0.002	3.38 (2.05–5.57)	< 0.001	2.45 (1.46–4.09)	0.001
Sarcomatoid feature (yes vs no)	5.53 (2.01–15.2)	< 0.001			6.55 (2.37–18.1)	< 0.001	3.27 (1.14–9.37)	0.028
SII (> 529 vs < 529)	1.92 (1.19–3.08)	0.007	1.78 (1.1–2.87)	0.018	1.91 (1.14–3.19)	0.013	1.8 (1.07–3.03)	0.027

## Discussion

In the present study, we explored the prognostic value of preoperative SII in non-metastatic RCC patients who underwent nephrectomy. We observed that high SII is likely to be associated with adverse factors, such as age, tumor size, pathological T stage, tumor grade, and necrosis. Multivariate analyses showed SII was an independent predictor of OS and CSS. Moreover, to limit the influence of bias, we performed PSM and demonstrated that high SII was also an unfavorable factor for OS and CSS in the matched cohort.

The association between inflammation and malignancy has been widely explored in the past decades. Immune cells play an essential role in the inflammatory process leading to the production of cytokines and chemokines that promote tumor development, invasion and metastasis [8]. Therefore, the complex balance between immune cells and substances secreted by inflammation may affect the type of cells detectable in the peripheral circulation. SII, a novel inflammation index, based on the proportion of neutrophil, platelet, and lymphocyte, was found to be associated with the prognosis of several malignancies. A retrospective multicenter cohort study revealed that high SII (> 900) is associated with poor CSS (HR = 2.32; 95% CI 1.55–3.48) in patients with resectable pancreatic cancer [22]. Chen et al. included 1383 colorectal cancer patients and found high SII (> 340) was an independent predictor of OS and disease-free survival [23]. Zhang et al. conducted a retrospective analysis of 209 patients with BC undergoing radical cystectomy, finding SII was an independent predictor for OS and provided more accurate prognostic predication than neutrophil-to-lymphocyte ratio (NLR), platelet-to-lymphocyte ratio (PLR), and C-reactive protein/albumin ratio [17]. Similarly, Jan et al. observed that SII is superior to NLR, PLR and monocyte-to-lymphocyte ratio (MLR) as a predictor of survival in patients with UTUC [24]. Besides, a high SII was associated with adverse characteristics such as lymphovascular invasion, positive lymph node and more aggressive phenotype, which is consistent with our findings.

Similarly, SII was also reported to be associated with survival outcomes in patients with RCC. Chrom et al. involved metastatic RCC (mRCC) patients treated with the tyrosine kinase inhibitor and found that high SII was an independent factor for inferior OS [25]. Besides, they found that the addition of the SII to the International Metastatic Renal Cell Carcinoma Consortium (IMDC) model in place of neutrophil and platelet counts increased the model’s prognostic performance. Lolli et al. also included patients with mRCC treated with sunitinib and observed SII was associated with the OS [18]. To be noted, the improvement of SII value at 6 weeks (from ≥ 730 to < 730) was correlated to a better prognosis, as a possible effect of sunitinib on peripheral blood cells secondary to decline in inflammation process [18]. De Giorgi et al. suggested that SII appears superior to the NLR as a prognostic marker in mRCC patients treated with immune check-point inhibitors [26]. Furthermore, recently, a retrospective study of 176 RCC patients who underwent radical nephrectomy observed that high SII was associated with poor OS (P = 0.034) [27]. SII is also associated with an increased TNM stage, which is similar to our results. However, no significant association was found for CSS (P = 0.29). Although their study was limited by the small number of patients and the short duration of follow-up, it was valuable for exploring the prognostic value of SII in the non-metastatic RCC. To the best of our knowledge, there are no other studies focusing on the prognostic impact of SII in surgically treated non-metastatic RCC patients. There is no standard cut-off value of SII, and the cut-off value of SII differs from study to study. Hence, the multicenter study is required to identify standard cut-off value.

The potential mechanism for the prognostic significance of this combination might be explained by the functions of neutrophil, platelet, and lymphocyte. Neutrophils promote angiogenesis and inhibit anti-tumor immune system response, leading to tumor development [9, 12, 28]. Neutrophils also play an important role in the lymphangiogenesis [29]. Furthermore, neutrophils could secret circulating growth factors such as vascular endothelial growth factor, facilitating adhesion and tumor seeding [29, 30]. Huang et al. Observed that neutrophilia is an independent predictor of recurrence in patients with RCC [31]. Besides, the intratumoral neutrophil is associated with poor prognosis in patients with localized and metastatic RCC [32, 33]. Platelet could protect circulating tumor cells (CTCs) during circulation, induce CTC epithelial-mesenchymal transition, and facilitate the extraction of tumor cells, leading to the metastasis of tumor cells [34]. Moschini et al. revealed that the platelet count was associated with survival in patients with bladder carcinoma [35]. Yun et al. also found that decreased mean platelet volume was independently associated with RCC [36]. Lymphocyte plays an important role in anti-tumor immunity. Lymphocyte could induce cytotoxic cell death and inhibit tumor proliferation and migration by secreting cytokines, leading to a host immune response to malignancy [8]. A decreased lymphocyte count might result in the attenuation of immunological anti-tumor response. Lymphopenia was found to be associated with lower survival in patients with advanced bladder cancer and RCC [37, 38]. Besides, higher tumor-infiltrating lymphocytes mean more strongly anti-tumor effect and better survival outcomes [39]. Therefore, the high SII, which reflects thrombocythemia, neutrophilia, or lymphopenia, suggests weak adaptive immune response in patients. All of these could cause more tumor cells to escape from the host immune system, lead to an increase in circulating growth factors and tumor cells, and finally facilitate the tumor invasion and metastasis. Based on the above-mentioned evidence, high SII could serve as an unfavorable factor in cancer patients. However, more large scale studies are required to verify our findings.

SII had significance in clinical practice. SII was calculated based on the neutrophil, platelet, and lymphocyte, which is convenient, easily obtained and commonly tested before the treatment. SII could predict the prognosis of patients, which could be used for risk stratification. It could provide physicians with useful information and guide the treatment, adjuvant therapy and follow-up for patients. However, non-tumor associated inflammation might cause the change of SII. Similarly, the patients with some sort of deficiencies might have more risk of change of SII. Therefore, we need to consider these conditions and might provide supportive treatments. Besides, the individual conditions should be considered during the treatment strategy decision.

Our study is not devoid of limitations. Firstly, the present study is a retrospective study with potential selection bias. To limit the influence of bias, propensity score matching was conducted to balance the baseline. Secondly, the data was extracted from single hospital, and the sample size is moderate. A multicenter study with a large scale is further required. Next, although we excluded the patients with the presence of inflammation, other unknown conditions may have existed which affected the neutrophils, lymphocytes, and platelets. Finally, there is no standard cut-off value of SII. Therefore, we determined the cut-off value based on our data. More studies are necessary to identify the optimal cut-off value.

## Conclusion

In conclusion, preoperative SII is associated with adverse factors for RCC. Furthermore, higher preoperative SII is an independent predictor of poor OS and CSS in surgically treated patients with non-metastatic RCC. More prospective and large scale studies are warranted to validate our findings.

## Data Availability

The data used during the current study are available from the corresponding author on reasonable request.

## Abbreviations

RCC: Renal cell carcinoma
OS: Overall survival
CSS: Cancer-specific survival
HR: Hazard ratio
CI: Confidence interval
SII: Systemic immune-inflammation index
ROC: Receiver operating characteristic
